# Occupational Safety of Municipal Police Officers: Assessing the Vulnerability and Riskiness of Police Officers’ Work

**DOI:** 10.3390/ijerph18115605

**Published:** 2021-05-24

**Authors:** Viktor Soltes, Jozef Kubas, Andrej Velas, David Michalík

**Affiliations:** 1 Department of Security Management, Faculty of Security Engineering, University of Žilina, 010 26 Žilina, Slovakia; andrej.velas@fbi.uniza.sk; 2 Department of Crisis Management, Faculty of Security Engineering, University of Žilina, 010 26 Žilina, Slovakia; jozef.kubas@fbi.uniza.sk; 3 Occupational Safety Research Institute, Jeruzalemska 1283/9, 110 00 Nové Město, Czech Republic; michalik@vubp-praha.cz

**Keywords:** occupational health, municipal police, police, risk assessment, vulnerability

## Abstract

The municipal police agencies increase the safety of a municipality’s citizens and thus increase their quality of life. When performing interventions, municipal police officers may endanger their safety and health. This paper deals with the analysis of the riskiness of municipal police officers working in the Slovak Republic and the Czech Republic from 2004 to 2019 and the assessment of their occupational safety. The risk analysis was carried out on the basis of a risk matrix and calculations of the probability of attack and injury to municipal police officers. Using the Pearson correlation coefficient, the dependence between the selected variables was investigated. The reliability of this dependence was examined by the determination coefficient. The main result of the paper is the determination of the riskiness category of municipal police officer work based on the assessment of the occupational health protection of officers through statistical indicators of their activities and risk matrix. The results will serve as part of the explanatory memorandum for the proposal of legislative changes in order to increase the occupational health protection of municipal police officers.

## 1. Introduction

Ensuring the internal security of the state and public order is one of the basic tasks of the state. The fulfilment of this task is in the competence of state security agencies, whose activities are supplemented by others, especially private security agencies. Among the key security agencies whose task is to ensure the internal security of the state and public order, most belong to police security agencies. In the conditions of Slovakia, there are two police agencies, which differ from each other in territorial scope, competencies, structures, and other aspects. The Police Force of the Slovak Republic (hereinafter referred to as the Police Force) is the primary armed security agency with nationwide competence, which performs tasks in matters of internal order, security, and fighting against crime, including its organized and international forms, as well as tasks arising from the international obligations of Slovakia [1]. The activities of this state police security agency are complemented by another non-state police security agency. Municipal police agencies can be considered as non-state police security agencies, which are order-keeping agencies operating in ensuring municipal public order, environmental protection in the municipality, and performing tasks arising from generally binding municipal regulations, municipal council resolutions, and decisions of the mayor [2]. Municipal policing can also be considered as the realization of “incorporated local authority duties, which included crime prevention and patrol by a variety of local enforcement agents, normally employed and controlled by municipal authorities and paid out of public funds, but not always sworn police officers” [3].

The municipal police agencies perform tasks only in a defined territory, so their competence is not nationwide. Their police activity is converging to the police activity performed by the Police Force, as is the case in many member states of the European Union [4,5,6]. On the contrary, some member states of the European Union do not use the services of the municipal police in their metropolises [7].

The municipal police agency is established and abolished by the municipality via a generally binding municipal regulation. Given that the municipality is an independent, territorial, self-governing, and administrative unit of Slovakia, no one can order it to establish a municipal police agency, and, therefore, not every municipality has such a security agency [8,9]. The reason for the non-establishment of the municipal police agency is mainly the lack of funds necessary for its operation. However, the effectiveness of police agencies does not always depend on the amount of money invested in security [10]. Other literature also deals with the assessment of the cost and effectiveness of municipal police [11,12,13].

Some authors point out the importance of the education and training of police officers [14,15,16]. In the conditions of Slovakia, the municipal police officers are regularly retrained in order to increase their legal awareness. In the case of mental health protection, some police agencies in Europe have implemented training programs aimed at managing stressful situations occurring in the performance of the police officer profession [17].

The municipal police may realize its powers only within the limits of the Municipal Police Act (1991), which defines its basic tasks and obligations [2]. Due to the nature and scope of the tasks performed by the municipal police agencies, their officers have the status of public officials in the performance of their tasks, as well as members of the Police Force. They are therefore law-protected persons, and an attack on such persons committed in connection with the authority and responsibility of the officer is considered a criminal offense. Based on that fact, there is an increased risk of an accident at work when performing the tasks of the municipal police compared to other types of professions. From the current practice, the most common causes of violence perpetrated on police officers include the uncertainty of a police officer during an intervention, the mental unpreparedness of a police officer to cope with a stressful situation, underestimation of the situation by a police officer, denigration of police officers in the media, and unfavorable public opinion of some citizens about the police [18].

According to the level and nature of the work, work environment factors that may affect the health of employees, health risk assessments, and changes in the health status of employees, works are classified into four categories according to the Protection, Promotion and Development of Public Health Act (2007) [19].

Works in which the limits of work and work environment factors set by special regulations are not exceeded are included in Category 1 and Category 2. These works can be described as non-hazardous. There is no risk or presumption of damage to health, but, at the same time, it is not possible to completely rule out the adverse consequences of the workload that affects the employee (stress, eye strain when working with a computer, long-term sitting, etc.) [19].

Works classified in Category 3 and Category 4 are considered as hazardous, because they generally exceed the limits of work and work environment factors set by special regulations [20]. Hazardous work can be defined as work in which there is an increased risk of developing an occupational disease, professional poisoning, or other damage to health in connection with the work [19]. The classification of a specific work into Category 3 and Category 4 or its exclusion is decided by the relevant Regional Public Health Authority of the Slovak Republic (according to the seat of the employer), either on the basis of the employer’s proposal or on its own initiative.

Determining the risk category of any work performed in Slovakia is individual, given that persons performing the same job position within different employers may perform different types of work tasks. The work tasks performed by municipal police officers are determined by generally binding regulations, and are uniform for all municipal police officers. For this reason, it would be appropriate to set a minimal risk category for the work of municipal police officers. The research objective is therefore to assess the occupational safety of municipal police officers on the basis of statistical indicators (on the number of attacks against officers and the number of their injuries) and on the basis of an analysis of work and work environment factors by risk matrix.

## 2. Materials and Methods

Statistical data were required to carry out a detailed overview study of the activities carried out by municipal police officers, which will make it possible to identify the vulnerability and thus the riskiness of this profession.

### 2.1. Research Design

In order to increase the occupational safety of municipal police officers, it is necessary to analyze in detail the basic statistical indicators of their activities. The initial regional analysis should determine the coverage of the territory in which the municipal police agencies operate. The territorial scope of municipal police agencies is a starting point for further understanding of the research context. Subsequent analysis of basic statistical indicators on the number of interventions, the number of recorded offenses, and the number of coercive measures used can monitor the development of the activities of municipal police agencies, which may have an impact on the occupational safety of municipal police officers. The probability of an attack on municipal police officers and the probability of their injury will be calculated on the basis of simple formulas that have already been used in the past, for example, in Czechia. Their benefit is their easy use in practice to quickly examine the current state of occupational safety of police officers. The relationship between the number of police officer injuries and the number of attacks against them will be investigated using the Pearson correlation coefficient. Verification of the achieved results can be realized by comparing them with the results of examining the same indicators in Czechia, where municipal police agencies work on the same basis as in Slovakia. The last part of the research is the assessment of risk factors of the work of municipal police officers on the basis of generally binding regulations and a risk matrix, through which the risk category of police officers’ work is determined.

In order to classify employees into individual risk categories, it is necessary to pay attention to the factors that influence their classification. A decree on the details of factors of the work and work environment in relation to the categorization of the work in terms of health risks and requisites of the proposal for the categorization of works (2007) sets out details on work and work environment factors for the categorization of works. These factors include: noise, vibrations, electromagnetic field, ultraviolet radiation, infrared radiation, lasers, intense pulsed light, ionizing radiation, increased air pressure, chemical factors, carcinogenic and mutagenic factors, biological factors, heat load, cold load, physical load, and mental workload [21].

Several experts have dealt with the assessment of some work and work environment factors in their scientific works and studies. Arjunan and Rajan (2020) pointed to the problem of increasing noise due to urbanization and car use. In their study, they focused on the risk associated with long-term exposure in a noisy environment. This has a negative effect on the auditory system, but also on the nervous system itself [22]. Another risk is associated with infection during work. This fact is supported by the research carried out in Poland, where the authors pointed out the importance of keeping a distance from the persons for whom officers are performing the intervention as a possible prevention of infection [23]. Municipal police officers in Slovakia and Czechia perform similar work tasks as in Poland, and, therefore, it is possible expect similarity in daily meetings with people. While clarifying the offenses, the municipal police officer has to legitimize the person, and, therefore, it is not always possible to follow the required distance. Other factors related to the weather have an impact not only on health, but also on the work performance of police officers [24]. Due to warm weather, a higher number of police officer injuries at work was recorded [25]. However, cold weather also has a number of other negative effects on the police officer’s work. Working in cold weather threatens the immune system and causes damage to the skin due to frostbite. Cold weather acts on the body, which can contract blood vessels and thus reduce blood flow. This reaction may increase the risk of heart attack [26]. In terms of physical load, physical force is also used by police officers to perform work tasks. During interventions, there is an increased risk of an accident at work, as well as the risk of injury to the person against whom the intervention is directed [27]. Some studies also confirm problems related to the mental workload of police officers; therefore, it is necessary to focus on assessing such effects [28].

### 2.2. Participants and Sampling

The research sample is represented by statistics of the municipal police officers’ activities in Slovakia. Monitoring of municipal police has been performed by the Ministry of the Interior since 2004. In 2004, a total of 152 municipal police agencies operated in Slovakia, which represents 5.26% of the total number of 2890 municipalities in Slovakia. In that year, 2192 officers worked in these municipal police agencies. According to Matusiak (2019), the number of police officers is influenced by a number of factors, which may include, for example, the population of the community, the territorial size of the jurisdiction, and demography [29]. The number of municipal police agencies in the first half of the reference period (from 2004 to 2010) increased, which is also related to the growth of municipal police officers. Since 2008, the number of municipal police agencies has oscillated around 165, and, in 2012, for the first time in history, the number of municipal police officers exceeded 2500. Giblin & Nowacki (2018) in their research dealt with the effects of the global economic crisis that occurred in 2008–2014 on police agencies. Based on the analysis of municipal police agencies in Slovakia, it can be stated that the global economic crisis had almost no effect on them [30]. In 2019, a total of 2547 officers worked in 167 municipal police agencies in Slovakia. This is the highest number of municipal police agencies, but also the highest number of municipal police officers in the history of independent Slovakia.

To verify the research results on occupational safety of municipal police officers in Slovakia, their comparison with the results of the analysis of occupational safety of police officers in Czechia was used. The research sample for Czechia is represented by statistical indicators on the activities of municipal police officers operating in their territory. The number of municipal police employees in Czechia has been above 9500 since the beginning of the reference period. The highest number of 9806 employees was recorded in 2016. Since this year, the number of municipal police employees has fallen to 9658 for three consecutive years. The majority of employees are municipal police officers, however, since the beginning of the reference period, it has been possible to observe a steady decline (while in 2010, there were 8613 municipal police officers, in 2019, there were only 8288).

### 2.3. Information Collection

The Ministry of the Interior annually publishes statistics on the municipal police activities in Slovakia for the previous calendar year and provides data on the number of municipal police officers, complaints, performed interventions, coercive means use, attacks and injuries of municipal police officers, solved offences, etc.

Assessing the vulnerability of municipal police officers and thus determining the riskiness of their work did not require a detailed analysis of all above data. To verify the results, a similar analysis was appropriate to carry out in another country, where municipal police operate with almost identical tasks, competencies, and authorizations. Such a country in which the municipal police carry out their activities on the same principle as the municipal police in Slovakia is Czechia. Municipalities that have established municipal police are obliged to send to the Ministry of the Interior of Czechia statistical data of the municipal police activities for the previous calendar year. Members of the municipal police in Czechia are divided into three categories. These are officers, candidates, and other employees [30]. Although such a division of members of the municipal police is not used in Slovakia, in other countries, such a division is common practice [31,32,33,34]. In Poland, for example, members of the municipal police can be divided into three groups according to the tasks they perform: prevention, performance, and logistics [35].

Municipalities are obliged to provide the Ministry of the Interior of Czechia, upon request, information on the number of candidates, officers, and other employees of the municipal police, age structure and qualifications, financial costs for the activities of the municipal police, the number of solved offenses, the number of cases of weapon use by officers, the number of attacks on officers, etc.

### 2.4. Data Analysis Strategy

The statistics on municipal police collected in Slovakia and Czechia are different, and, therefore, the comparison of municipal police activities in these two countries was only partially possible. However, in terms of the riskiness of municipal police officer work, it was possible to compare data on the number of attacks against the municipal police officers and thus determine the probability of an attack on them, which can be calculated according to Formula (1) [36]:

Probability of attack = [(number of attacks)/(number of officers)] × 100,(1)

In the conditions of Slovakia, in addition to the probability of an attack on a municipal police officer, it is also possible to calculate the probability of injury (vulnerability probability) of municipal police officers against whom the attack was conducted according to Formula (2) [36]:

Probability of injury (vulnerability) = [(number of injuries)/(number of attacks] × 100,(2)

Vulnerability assessment can also be performed on the basis of other, more complex methods. One of the most widely used methods for measuring vulnerability is the AHP method [37]. Several methods are originally designed to measure vulnerability in the economic sciences, where the Risk + Response = Vulnerability rule is used. Such methods include, for example, the Progress out of Poverty Index (PPI) or Poverty Assessment Tools (PATs). By modifying some methods for measuring vulnerability in the economic sciences, their use can be extended to the area of livelihood. For example, the Household Vulnerability Index (HVI), the Local Vulnerability Index (LVI), or the Participatory Rapid Appraisal (PRA) can be used in this area [38]. Although there are a number of other methods that can be used to assess vulnerabilities, they would be very difficult to use, given the available data, to assess the vulnerability of municipal police officers, and would not capture the substance of the research.

The calculation of the vulnerability probability according to Formula (2) is based on the hypothesis that there is a relationship between the number of attacks on municipal police officers and the number of their injuries. This hypothesis can be verified by calculating the Pearson correlation coefficient between these two variables. The correlation coefficient can take values in the range <−1.1>. If the correlation coefficient is equal to 0, it means that there is no linear relationship between the investigated variables. If the correlation coefficient is equal to 1, then the variables are directly dependent on each other; an increase in one variable causes an increase in the other. If the correlation coefficient is equal to −1, an increase in one variable causes a decrease in the other.

The reliability of the model can be expressed based on the coefficient of determination, which is defined as the square of the correlation coefficient. The interpretation of the coefficient of determination is based on the analysis of the variability (variance) of the dependent variable (number of injuries), which should be largely explained by the variability of the independent variable (number of attacks), assuming that the magnitude of the values of the first variable depends on it linearly.

Based on the determination of the probability of attacks and the vulnerability probability of municipal police officers, it is possible to point out the riskiness of this profession. In the conditions of Slovakia, explicitly determined factors are currently used to determine the riskiness of work, on the basis of which works are included in one of the four risk categories. However, it is not possible to assess the riskiness of specific professions by these factors (due to the scope and nature of their work tasks), among which it is possible to include municipal police officers. In addition, the insufficient classification of individual works into the risk category according to individual factors does not consider the outbreak of a pandemic, which affects working conditions in individual professions in the long term. In the case of armed security agencies, the vulnerability probability of their employees could also be taken into account when classifying them into one of the risk categories. This could increase the occupational safety of these employees, and thus increase the attractiveness and dignity of such professions. Therefore, it is necessary to focus on how individual factors endanger police officers during work.

Determining the risk of work and work environment factors of municipal police officers can also be carried out through qualitative methods, including a risk matrix, which evaluates two factors: the probability (likelihood) and consequences (severity) of risk. It allows to group risks into groups that prioritize corrective action to minimize existing hazards. Table 1 shows the values on the basis of which the assessment of the likelihood and severity of the identified risks was realized.

## 3. Results

The research results consisted of two basic parts. The first part contained a detailed analysis of municipal police activities. The analysis itself consisted of two parts: a regional analysis of the territorial scope of municipal police agencies and an analysis of basic activities related to the occupational safety of municipal police officers, which was used to calculate the probability of attack against them and the probability of their injury. The second part of the research is the assessment of work risk factors that determine the risk category of municipal police officers.

### 3.1. Statistical Assessment of Municipal Police Officer Activities in the Context of Work Riskiness

**Figure 1:**
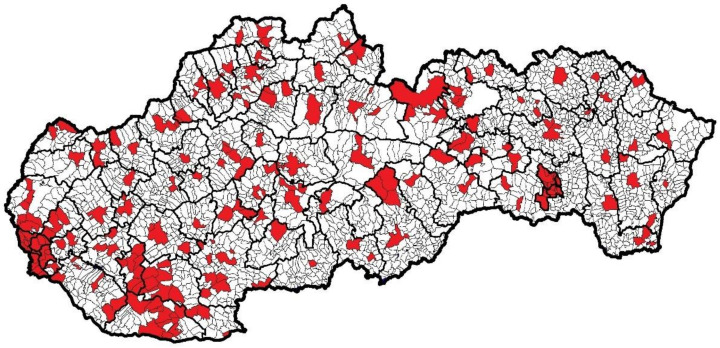
The territory of Slovakia covered by municipal police agencies (marked with red).

Municipal police officers perform their tasks only in the territory of the municipalities by which they were established. Figure 1 shows the territories of Slovakia in which municipal police agencies perform their tasks.

Pursuant to the Decree of the Statistical Office of the Slovak Republic issuing the classification of statistical territorial units (2004), the territory of Slovakia is hierarchically divided into three regional levels (NUTS 1, NUTS 2, and NUTS 3) and two local levels (LAU 1 and LAU 2). NUTS level 1 represents the territory of all of Slovakia, NUTS level 2 represents territories of four areas, NUTS level 3 represents eight higher territorial units, LAU level 1 represents 79 districts, and LAU level 2 represents the territory of 2927 municipalities and city parts.

The territory of Slovakia that is under the jurisdiction of municipal police agencies makes up a total of 8267.38 km^2^, which represents 16.86% of the entire territory of Slovakia. From the point of view of NUTS level 3 regions, the largest coverage area is in the Bratislava region (40.69%) and in the Nitra region (27.44%). Other NUTS level 3 regions are covered to less than 20%.

The number of inhabitants who are under the protection of municipal police officers in Slovakia represents 2,978,212, which is 54.57% of the total population. In terms of NUTS level 3 regions, most inhabitants of the Bratislava region (82.57%) and the lowest number of inhabitants of the Trnava region (44.21%) are under the control of municipal police officers.

According to several experts, in the conditions of Slovakia, the optimal ratio would be a thousand inhabitants of the territory per one municipal police officer. Within the entire territory of Slovakia in which the municipal police agencies operate, one municipal police officer cares for an average of 1170 inhabitants, which is a slight deviation upwards from the optimal ratio. In terms of NUTS level 3 regions, the worst in this count is the Bratislava region (1386 inhabitants per one municipal police officer); on the contrary, the best is the Trnava region (1015 inhabitants per one municipal police officer). None of the NUTS level 3 regions has had the proportion of the population per one municipal police officer fall to less than 1000:1.

**Figure 2:**
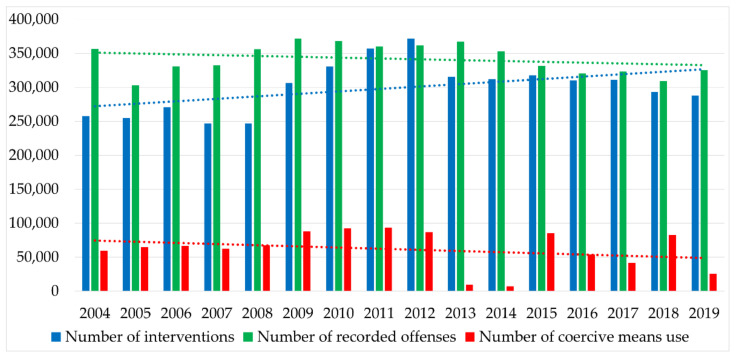
Basic statistical data of municipal police agencies in Slovakia [41].

The success of police task performance resulting from generally binding regulations depends primarily on the success of interventions. Resolving offenses and other antisocial activities by municipal police officers in many cases requires the use of coercive means. The municipal police officers may use coercive means only in exhaustively defined cases, when the person against whom the intervention is directed does not cooperate, or when the municipal police officer or a third party is in imminent danger to life or health. The use of coercive means increases the risk of property damage or injury or death to the person against whom the intervention is directed, or to an uninvolved person. Figure 2 shows the statistics of performed interventions, using coercive measures and recorded offences by municipal police officers in the period 2004–2019.

The number of interventions performed by municipal police officers per year is, on average, around 300,000. The largest number of interventions was recorded in 2012 (371,734 interventions), and the smallest in 2008 (246,454 interventions). In the last three years of the reference period, a decreasing number of interventions was recorded.

As already mentioned, municipal police officers are also entitled to use adequate and statutory coercive means during the intervention if the nature of the intervention so requires. In the individual years of the reference period, the number of cases when coercive means were used jumped, and it is not possible to determine a clear trend of development. The problem with the evaluation of this indicator lies in the ambiguous interpretation of the reporting of the technical means used to prevent the departure of a vehicle used by municipal police officers. It was in 2013 and 2014 that these technical means were not recorded by municipal police officers as coercive means, which reduced the total number of coercive means used to less than 10,000. Among all coercive means, it is possible to include the use of a weapon or the use of a warning shot as the riskiest coercive means. With the abovementioned coercive means, it is possible to observe a decrease in the reference period. While, at the beginning of the reference period, in 2004, 90 cases of weapon use were recorded, in 2019, only three such cases were recorded. The use of warning shots as coercive means in the first half of the reference period ranged from five to 10 cases; in the second half of the reference period, the number of such cases decreased to less than five cases.

The intervention of municipal police officers can also result in injuries and, in fatal cases, in the death of persons, whether it is the person against whom the intervention is directed, an uninvolved person, or the municipal police officer him or herself. On the positive side, the activities of municipal police officers in the last 16 years did not result in the death of any person or in the injury of an uninvolved person. However, injuries were recorded in persons against whom the intervention was directed. The total number of cases in which the person against whom the intervention was directed was injured was 146 persons for the entire reference period. While, between 2004 and 2011, the number of injured persons against whom the intervention was directed each year (with the exception of 2006) exceeded 10, after 2011, more than 10 such persons were injured in 2014 alone.

The activity performed by the municipal police officers may not be accepted with understanding by citizens who have committed an offense or other antisocial activity unknowingly, and they have been sanctioned for this act, which they do not consider adequate. Dissatisfaction with the activity of municipal police officers may also be expressed by a group of the population who knowingly committed an offense or other antisocial activity, but consider the sanction imposed for such an act to be disproportionate. A separate group of the population who may not be satisfied with the activities of municipal police officers are people under the influence of alcohol, drugs, or other narcotic and psychotropic substances, aggressive people, and people with mental behavior disorders. These three groups of the population are willing and able to cross the boundaries of appropriate behavior and attack municipal police officers in defending their own interests. In such cases, however, they are unaware of the consequences of their actions, as the municipal police officer has the status of a public official during the performance of his or her tasks. According to the Criminal Code (2005), a person who uses violence with the intention of acting to the exercise of the authority of a public official or for the exercise of the authority of a public official has fulfilled the criminal offense of attacking a public official and shall be punished by imprisonment for at least one to five years [42]. Table 2 shows the number of attacks against municipal police officers and the number of cases in which they were injured. The table also contains the probability of an attack on one municipal police officer and the probability of injury due to the number of attacks (vulnerability probability).

The number of attacks on municipal police officers has been gradually declining since the beginning of the reference period. This decline may be caused by the development of society after socio-political changes related to the establishment of an independent state and the creation of security services in Slovakia. Citizens initially confused the concept of democracy with anarchy did not show respect for generally binding regulations, and were reluctant to cooperate with law enforcement agencies [43]. By improving the socioeconomic situation, citizens have gradually become accustomed to the status of state police agencies. In 2005, a new Criminal Code (2005) came into force, tightening penalties for attacks on a public official [42]. The Private Security Act was also entered into law in 2005, establishing a new status for private security services in which more and more people found employment [44]. Due to the improving socioeconomic situation and sociopolitical situation, as well as changes in generally binding regulations, the public’s view of municipal police officers has changed, and, despite the increasing number of municipal police officers, the number of attacks against them has decreased.

**Figure 3:**
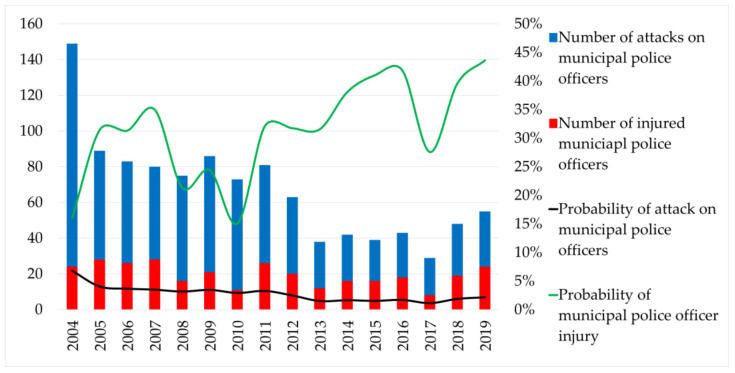
Vulnerability of municipal police officers [41].

Most attacks on municipal police officers were recorded in the first year of the reference period, in 2004, when up to 149 attacks were recorded. This means that 6.8% of municipal police officers were attacked that year. Gradually, the number of attacks, and thus the share of attacks given one officer, decreased. The best situation, and therefore the least attacks on municipal police officers, was recorded in 2017 (29 attacks, 1.15% of municipal police officers). Over the next two years of the reference period, the number of attacks began to rise again. In 2018, 48 attacks were recorded, and, in 2019, up to 55 attacks were recorded, which means that the share of attacks on officers exceeded 2% after seven years. In connection with attacks against municipal police officers, the number of their injuries was also related. The number of injuries suffered by municipal police officers in the performance of their work is developing in a similar way to the number of attacks directed at them. The highest number of attacks was recorded in the years 2004–2007, in 2011, and in the last year of the reference period, in 2019. It was in 2019 that the number of injuries of municipal police officers reached the highest value given the total number of attacks directed against them. Out of all 55 attacks against municipal police officers, up to 24 injured officers were recorded, representing a 43.64% share. Figure 3 shows the number of recorded attacks against municipal police officers, the number of their injuries, the probability of an attack given the total number of officers, and the probability of injury of a municipal police officer given the total number of attacks.

**Figure 4:**
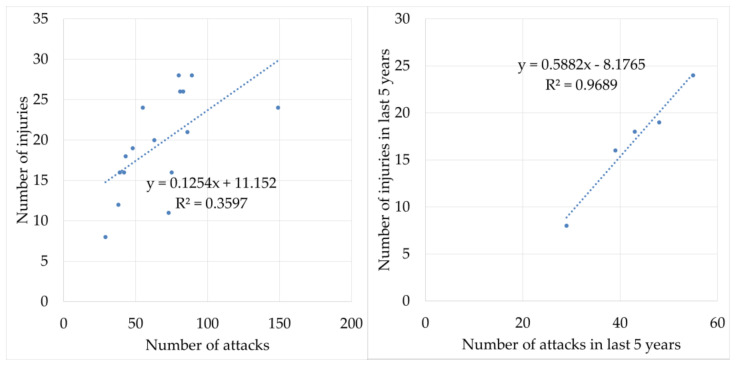
Dependence of variables related to the vulnerability of municipal police officers. y—regression equation; R^2^—coefficient of determination.

The number of injured municipal police officers was correlated with the number of attacks directed against them. The Pearson correlation coefficient for the whole reviewed period reached the value of 0.6, which indicated a slight dependence between the examined variables. The value of the correlation coefficient between the number of attacks and the number of injuries of municipal police officers in the last five years of the reference period even increased to 0.98, which indicated a very strong linear dependence between the examined variables. The reliability of the model can be expressed by the coefficient of determination, the value of which for the whole monitored period reached only 0.36, but for the last five years, the value was equal to 0.97. This fact can be interpreted as the fact that the number of injuries of municipal police officers can be 97% explained by the number of attacks directed against them. Figure 4 shows the dependencies of the variables.

To verify the achieved results of the probability of attacks on municipal police officers in Slovakia, and thus the riskiness of their work, it was necessary to realize an analysis of attacks on municipal police officers in Czechia, and then compare these results.

The Czech Republic Municipal Police Act (1991) states that the municipal police ensure local matters of public order within the competence of the municipality and perform other tasks, if so, provided by this act or a special law.

The tasks, duties, authorizations, and coercive means used by municipal police officers in Slovakia and in Czechia are almost the same, and differ only minimally. This fact makes it possible to make a comparison between these police security forces performing their tasks in two different countries.

The analysis of attacks directed against municipal police employees in Czechia should be realized only with the given number of municipal police officers, not the total number of municipal police employees, as a group of candidates and other employees do not fully perform their tasks in the streets. Table 3 shows the number of municipal police employees in Czechia by work category, the number of attacks on municipal police officers, and the probability of attacks on municipal police officers in 2010–2019.

Given the number of officers, it can be stated that the number of attacks on municipal police officers in Czechia is higher than the number of attacks against municipal police officers in Slovakia. In parallel, the probability of an attack on one municipal police officer in Czechia is higher than the probability of an attack on one municipal police officer in Slovakia. The probability in Slovakia decreased until 2017, and approached almost 1%, but, in 2018, it slightly increased to almost 2%. In Czechia, the probability of attacks on municipal police officers is decreasing in the long term. From a probability of 6% in 2012, this probability decreased, and, in 2019, it reached 2.75%.

### 3.2. Assessment of Municipal Police Officer Work Riskiness According to the Work and Work Environment Factors

The classification of individual types of work into a specific risk category is nowhere precisely established. Every employer, in cooperation with the occupational health service, must ensure the categorization of work performed at the workplace. Table 4 shows the assessment of the risk of work of municipal police officers according to the work and work environment factors.

Based on factor A, noise, most municipal police officers would be in Category 1, with the exception of municipal police officers in large cities. In the capital of Slovakia, measurement was performed, where the average values of noise were measured in the morning peaks up to 92.5 dB, in the afternoon 81.6 dB, and in the evening 76.1 dB [49]. Based on this, it would be possible to classify municipal police officers in Category 2.

Factors B, C, D, E, F, G, H, I, J, and K do not apply to the work of municipal police officers, or they encounter these factors only in exceptional cases. For this reason, the individual categories for these factors are not determined.

In assessing the L, biological factors, it is necessary to take into account the fact that municipal police officers come into contact with people in the operation of their profession. In certain circumstances, such as in an attack against them, municipal police officers are also entitled to use coercive means. In an attack on a municipal police officer, the attacker can also use various forms of weapons. If the attacker is infected with an infectious disease, there is a risk that such an attack could also infect the municipal police officer, and, therefore, the municipal police officers could be included in Category 2. Classification in other categories can only be considered in exceptional cases, including the pandemic associated with COVID-19. Even in such a specific situation, when there is an increased risk of encountering infected persons with COVID-19 and subsequent infection from these persons, municipal police officers must perform their duties.

Municipal police officers perform their work tasks primarily outdoors. Due the fact that, in the summer, the average temperature in Slovakia is higher than 13 °C, according to the factor M, heat load, they can be included in Category 2 on the basis of the decree on details of health protection against heat and cold stress at work [47]. This decree, among others, defines an extremely warm day as the day on which the outside air temperature measured in the shade reaches a value higher than 30 °C. The highest measured temperature in Slovakia was on 20 July 2007 in Hurbanovo, 40.3 °C. The highest average annual temperature in 2019 was in Žihárec, and there was 13.1 °C. The highest monthly average temperature was recorded in August 1992 at the Bratislava housing estate Petržalka, 26.0 °C [50].

In the case of factor N, cold load, it is possible to classify municipal police officers in Category 2. The decree on details of health protection against heat and cold stress at work (2016) also defines an extremely cold day as the day on which the outside air temperature measured in the shade reached a value lower than −15 °C. The lowest temperature in Slovakia was measured on 11 February 1929 in Vígľaš, a part of Pstruša, −41.0 °C. The lowest measured temperature in the capital of Slovakia, Bratislava, in the city part of Devínska Nová Ves, was recorded on 11 February 1929, −36 °C. The lowest average annual temperature in Slovakia was recorded in 1956 on Lomnický štít, −5.6 °C. In the same year and at the same place, the lowest monthly average temperature was measured in February, −18.1 °C [50].

According to factor O, physical load, it is possible to include municipal police officers in Category 2. During walking activity, which they perform during their work tasks, they expend a relative amount of energy. Another fact on the basis of which it would be possible to include municipal police officers in this category is that, in the case of intervention using coercive means, they are forced to expend more energy in the short term than workers who fall into Category 1. The individual permissible values of physical activity for sex and age categories are specified in more detail by the decree on details of health protection against physical stress at work, mental workload, and sensory stress at work [48].

In the factor P, mental workload, it is necessary to focus on the decree on details of health protection against physical stress at work, mental workload, and sensory stress at work (2007), which evaluates mental workload in four levels according to eleven characteristics of the work and work environment: work intensity and time pressure, forced work pace, monotony, effects disrupting concentration, social interactions, material and organizational responsibility, risk of endangering the life and health of oneself or other persons, shift work, work environment, physical discomfort, and other sources of stress [48]. Tomei et al. (2018) in their work deal with the stress that arises in the work of municipal police.

If at least one characteristic corresponds to Category 3, but at most one to Category 4, then the work should be included in Category 3 [51]. In the case of municipal police officers, it is necessary to focus on the characteristics of the risk of endangering the life and health of oneself or other persons. Municipal police officers may be attacked and hurt while performing their profession and their duties.

Based on the assessment of individual work and work environment factors of municipal police officers in accordance with the decree on details of factor of work and the working environment in relation to the categorization of work in terms of health risks and requisites of the proposal for categorization of works (2007), it is possible to include their work in Category 2 of risk [21]. Taking into account the current global situation related to the airborne COVID-19 pandemic, which poses an increased risk of infection for municipal police officers, their profession could be classified in Category 3 according to L, biological factors. If at least one work and work environment factor for the classification of works is included in Category 3, all workers performing this work are included in Category 3 of risk.

Identified work and work environment factors can be assessed through a risk matrix, which results in the prioritization of risks that need to be treated. Table 5 contains a risk matrix of identified work and work environment factors of municipal police officers.

Based on the occupational health risk matrix for Slovak municipal police agencies, biological factors can be considered the most serious risk. This is due to the current situation related to COVID-19. After treating the situation associated with this disease, it can be assumed that the likelihood will decrease to an unusual level. This risk would then reach the high level, but this work and work environment factor would still need to be addressed.

## 4. Discussion

Based on the analysis of statistical data on municipal police agencies operating in Slovakia and comparing the obtained data with data obtained from the analysis of statistics on municipal police agencies operating in Czechia, it is possible to verify the existence of dependence between the number of municipal police officers and the number of attacks directed against them. At the same time, the hypothesis that the number of municipal police officer injuries depends on the number of attacks against them was verified. Although the number of municipal police officers in Czechia is more than three times higher than in Slovakia, the number of attacks directed against municipal police officers in Czechia is, on average, more than seven times higher than the number of attacks directed against municipal police officers in Slovakia. The deterioration of the situation can also be noticed from an increasing probability of the risk of directing an attack against municipal police officers in Slovakia. In 2019, the probability of injury to a municipal police officer in Slovakia given the total number of attacks reached the highest value for the entire reference period. All of these partial results indicate a deterioration in the municipal police officer’s security situation and a high level of riskiness in this profession.

Based on the above facts, it can be concluded that the classification of municipal police officers in Category 2 of work riskiness cannot be considered adequate due to the scope and nature of the performed tasks by them. The fact that the municipal police officer has the status of a public official, and thus is a protected person in the performance of his duties and tasks, indicates that, in his or her work, he or she may find him or herself in conflict situations in which he or she may be endangered by an attack by the person against whom the intervention is directed.

Police officers are also exposed to physically demanding activities that endanger their health [51,52]. It is also important to pay attention to the psychological stress they encounter during their work. A number of authors point to the psychological burden posed to police officers during their work [53,54]. Mental workload must not be underestimated, as it may affect the police even after work [6,55,56]. Its underestimation can lead in the long run to burnout syndrome or other mental problems that negatively affect his or her quality of life [57,58].

It is also necessary to assess the mental workload of the police officers objectively. Assigning police officers to a higher risk category would create an obligation for regular preventive medical examinations performed by specialists [28]. Another reason for assigning police officers into a higher risk category is the emergence of post-traumatic stress disorders in police officers, which could have been caused by two types of (potential) traumatic events: physical danger to oneself or witnessing the harm of another. Attacks on police officers analyzed in this paper can be the trigger for mental health problems [28]. For this reason, the article focuses on the attacks of police officers and their injuries resulting from these attacks. One way to improve police preparedness for various stressful situations is to train them. That is why it would be appropriate for officers to complete various courses or other forms of education [59,60,61].

In connection with the outbreak of the global pandemic related to COVID-19, the riskiness of municipal police officer work has increased even more. Due to the fact that municipal police officers have to perform their tasks (patrolling and walking, performing interventions against persons, etc.) during a pandemic, there is an increased risk of infection with COVID-19, which spreads through the air [62,63,64]. This fact is confirmed also by an occupational health risk matrix elaborated for Slovak municipal police agencies. It is precisely this aspect of the increased probability of infection with a disease spread via free air that must be taken into account when assessing biological factors of work risks according to the decree on details of factor of work and the working environment in relation to the categorization of work in terms of health risks and on the requisites of the proposal for categorization of works (2007). Category 3 of work riskiness includes the basis of biological factors works, in which exposure of a biological factor of group 3 (may cause serious human disease and serious danger to workers; they may pose a risk of spreading in the population, with effective prophylaxis or treatment usually available); if the infections they cause are spread through the air under normal conditions and for which effective prophylaxis or treatment is available, the conditions and manner of performing the work pose an increased risk to the employee. If one of the work and work environment factors for the risk classification can be included in Category 3 (in this case biological factors), then the whole work is included in Category 3 of work riskiness [21]. Police officers are at risk for several diseases, and various measures need to be taken to reduce the likelihood of illness during work [23,65]. Reassignment of municipal police officers from Category 2 to Category 3 of work riskiness, at least for the duration of a pandemic of an airborne disease (such as the COVID-19 pandemic), could increase their safety and health at work.

## 5. Conclusions

Occupational safety policy applies to every single employee, including work performed in the public interest aimed at ensuring the protection of citizens’ lives, health, and property. Such works include the work of municipal police officers. Based on the assessment of the job position, each employee is classified into one of four categories of work, depending on the risk to health. When evaluating a job position, several factors are always taken into account that could affect the employee and thus contribute to the deterioration of health. Based on the analysis of statistical indicators of municipal police officers’ activities, calculations of the probability of attack and the probability of vulnerability of municipal police officers, and based on the assessment of work and work environment factors, it can be concluded that, in order to increase police officers’ occupational safety, it is appropriate to include them in Category 3 of work riskiness.

One of the advantages of the reassignment of municipal police officers from Category 2 to Category 3 of work riskiness is the increase in healthcare. Persons performing work classified in Category 3 of work riskiness must undergo a preventive medical examination at least once every two years (for works classified in Category 2, it is recommended to perform a preventive medical examination of employees every three years), and this examination may only be performed by a doctor or specialist in the field of occupational medicine and toxicology (for works classified in Category 2 of work riskiness, preventive medical examinations are performed by a general practitioner).

In addition, employees performing works falling into Category 3 of work riskiness are entitled to additional leave, supplementary pension insurance, and wage compensation for difficult work performance. By including the municipal police officers in this risk category, it would be possible not only to improve the prevention of work diseases, but also reduce the probability of their injuries during the performance of their tasks, which is very intensely dependent on the number of attacks directed against them. The research results are very important for the needs of legislative changes related to increasing the occupational safety of municipal police officers. The results of the regional analysis of municipal police activities are also beneficial for central state administration bodies, which can use them in a more effective deployment of forces and resources aimed at citizens’ protection. The methodology for assessing work and work environment factors will serve not only to prepare legislative changes in the field of occupational safety, but also for the Regional Public Health Authority of the Slovak Republic, which currently decides on the categorization of specific work into Category 3 and Category 4 of work riskiness.

## Figures and Tables

**Table 1:** Occupational health risk matrix [39,40].

Likelihood		Severity											
		Slight				Moderate				Extreme			
		Insignificant = 1	Minor Incident = 2		Minor Injury = 3	Health Damage = 4	Injury = 5		Multiple Injuries = 6	Serious Injury = 7	Fatal = 8		Multiple Fatalities = 9
Rare	Impossible = 1	1	2		3	4	5		6	7	8		9
	Remote = 2	2	4		6	8	10		12	14	16		18
	Unlikely = 3	3	6		9	12	15		18	21	24		27
Occasional	Possible = 4	4	8		12	16	20		24	28	32		36
	Unusual = 5	5	10		15	20	25		30	35	40		45
	Known = 6	6	12		18	24	30		36	42	48		54
Frequent	Likely = 7	7	14		21	28	35		42	49	56		63
	Usual = 8	8	16		24	32	40		48	56	64		72
	Certain = 9	9	18		27	36	45		54	63	72		81
**Risk**		Minimum		Low		Medium-low		Moderate		High		Extreme	

Colouring within the table represents level of risk.

**Table 2:** Attack and vulnerability probability of municipal police officers in Slovakia [41].

Year	Number of Attacks on Municipal Police Officers	Number of Injured Municipal Police Officers	Probability of Attack on Municipal Police Officers	Probability of Municipal Police Officer Injury
2004	149	24	6.80%	16.11%
2005	89	28	4.05%	31.46%
2006	83	26	3.67%	31.33%
2007	80	28	3.50%	35.00%
2008	75	16	3.17%	21.33%
2009	86	21	3.46%	24.42%
2010	73	11	2.93%	15.07%
2011	81	26	3.28%	32.10%
2012	63	20	2.49%	31.75%
2013	38	12	1.51%	31.58%
2014	42	16	1.66%	38.10%
2015	39	16	1.54%	41.03%
2016	43	18	1.70%	41.86%
2017	29	8	1.15%	27.59%
2018	48	19	1.89%	39.58%
2019	55	24	2.16%	43.64%

**Table 3:** Basic statistical data of municipal police agencies in Czechia [36].

Year	Number of Municipal Police Employees			Number of Attacks on Municipal Police Officers	Probability of Attack on Municipal Police Officers
	Officer	Candidate	Other		
2010	9582			2.93%	15.07%
	8613	114	855		
2011	9542			3.28%	32.10%
	8581	95	866		
2012	9538			2.49%	31.75%
	8580	59	899		
2013	9516			1.51%	31.58%
	8482	85	949		
2014	9536			1.66%	38.10%
	8444	58	1034		
2015	9691			1.54%	41.03%
	8515	109	1067		
2016	9806			1.70%	41.86%
	8459	90	1257		
2017	9769			1.15%	27.59%
	8431	64	1274		
2018	9717			1.89%	39.58%
	8386	55	1276		
2019	9658			2.16%	43.64%
	8288	63	1307		

**Table 4:** Attack and vulnerability probability of municipal police officers in Slovakia [21,45,46,47,48].

Factor of Work	Category	Characteristics of Work
A. Noise	1	(a) Works in which the normalized noise exposure level L_AEX_,_8h_ does not exceed 75 dB for 8 h, or the peak level C of acoustic pressure L_CPk_ does not exceed 130 dB. (b) Works in which the normalized noise exposure level in a 40 h week exceeds 75 dB, but does not exceed the upper action value of noise exposure (L_AEX_,_8h_, a = 85 dB and L_CPk_ = 137 dB).
B. Vibrations	-	Not determined.
C. Electromagnetic field	-	Not determined.
D. Ultraviolet radiation	-	Not determined.
E. Infrared radiation	-	Not determined.
F. Lasers	-	Not determined.
G. Intense pulsed light	-	Not determined.
H. Ionizing radiation	-	Not determined.
I. Increased air pressure	-	Not determined.
J. Chemical factors	-	Not determined.
K. Carcinogenic and mutagenic factors	-	Not determined.
L. Biological factors	2	Works in which exposure is a group 2 biological factor (may cause human disease and could be hazardous to employees, but is unlikely to spread to the population, and usually effective prophylaxis or treatment is available) or a group 3 biological factor (may cause serious human disease and serious danger to employees; they may pose a risk of spreading in the population, with effective prophylaxis or treatment usually available) if the infections they cause do not normally spread through the air and for which effective prophylaxis and treatment is available.
M. Heat load	2	(a) Works performed in the long term at an indoor workplace, where the permissible value of the operating temperature is exceeded due to heat load from technology, but the degree of heat load does not require the limitation of working time by adhering to long-term tolerable working time and short-term tolerable working time. (b) Works performed in the long term at an outdoor workplace during the warm period (the warm period is a period with an average daily outdoor air temperature of 13 °C and higher).
N. Cold load	2	(a) Works performed in the long term at an indoor workplace, where, due to technological reasons, the permissible value of the operating temperature is not reached, but the operating temperature in these areas is not lower than +4 °C. (b) Works performed in the long term at an outdoor workplace during the cold period (the cold period is a period when the average daily temperature for two consecutive days falls below 13 °C). (c) Works associated with the alternation of large temperature differences, especially in refrigerators or freezers, at a change frequency of more than 15 times per shift or at change intervals of less than 30 min.
O. Physical load	2	(a) Works predominantly dynamic, performed by large muscle groups in which: 1. The shift net energy expenditure does not exceed the average and permissible values for the age groups of men and women, but exceeds 0.85 times the average and permissible values for the age groups of men and women; 2. The minute net energy expenditure does not exceed the permissible values for the age groups of men and women, but exceeds 0.85 times the permissible values for the age groups of men and women, or; 3. The heart rate does not exceed the values of the average shift heart rate for the age groups of men and women, but exceeds 0.85 times the value of the average shift heart rate for the age groups of men and women. (b) Works performed by small muscle groups in which: 1. The permissible shift average values for predominantly dynamic work and for work with static components of work for men and women are not exceeded, but 0.7 times the permissible shift average values for predominantly dynamic work and for work with static components of work for men and women are exceeded; 2. The number of movements per shift or per minute depending on the duration of the contraction and the amount of force exerted as a percentage of Fmax of the relevant muscle group is not exceeded, but is exceeded 0.5 times the number of movements per change or per minute depending on the duration of the contraction and the amount of force exerted as a percentage of the Fmax of the relevant muscle group; 3. The permissible values of short-term muscle load due to predominantly dynamic work are not exceeded even in the short term, but are 0.7 times the permissible values of short-term muscle load due to predominantly dynamic work is exceeded, or; 4. The number of movements of the small muscle groups of the hand and fingers does not exceed the permissible values of the exerted force during the whole shift, but exceeds 0.5 times the permissible values of the exerted force during the whole shift. (c) Works predominantly static, performed by small muscle groups in which the average shift exerted muscular force does not exceed the permissible values, but exceeds 0.7 times the permissible values. (d) Works associated with the transfer of loads in which the weight of the hand-moved loads does not exceed: 1. Maximum weight during the whole shift, but exceeding 0.5 times the maximum weight during the whole shift; 2. The maximum weight of the load, but exceeding 0.2 times the maximum weight of the load set for men and 0.5 times the maximum weight of the load set for women. (e) Works performed predominantly in the basic working position in sitting, standing, or changing positions, with regularly occurring, conditionally acceptable working positions or unacceptable working positions during work, but the permissible values are not exceeded.
P. Mental workload	2	Work in which the mental workload assessed according to the characteristics of work and the work environment reaches an increased intensity according to the method specified in Annex no. 5 of the decree on details of health protection against physical stress at work, mental workload, and sensory stress at work.

L_AEX_—Normalized noise exposure level; L_CPk_—Peak sound pressure level C.

**Table 5:** Occupational health risk matrix for Slovak municipal police agencies.

Hazard	Likelihood	Severity	Risk
Noise	Unlikely	Minor incident	Minimum
Biological factors	Likely	Serious injury	Extreme
Heat load	Known	Minor injury	Low
Cold load	Known	Health damage	Moderate
Physical load	Possible	Injury	Moderate
Mental workload	Likely	Minor injury	Medium-low

## Data Availability

Data is contained within the article.
